# Supplementary material for: Modelling parameter uncertainty reveals bushmeat yields versus survival trade-offs in heavily-hunted duiker Cephalophus spp
Source: PLoS One. 2020 Sep 28;15(9):e0234595. doi: 10.1371/journal.pone.0234595 (PMC7521732; doi:10.1371/journal.pone.0234595)
Supplement: S1 File — (DOCX) [file pone.0234595.s001.docx]

# Modelling parameter uncertainty reveals bushmeat yields versus survival trade-offs in heavily-hunted duiker *Cephalophus* spp.

Tatsiana Barychka^1*^, Drew W. Purves^2¶^, E.J. Milner-Gulland^3^ and Georgina M. Mace^1¶^

Supporting Information

S1 Table. Duiker dataset. The data were used to estimate intrinsic rate of natural increase, $\boldsymbol{r}_{\boldsymbol{max}}$ and carrying capacity, $\boldsymbol{K}$. Population growth rates were estimated using Caughley and Krebs (C&K) [40] or Cole’s (C) [39] method (see S2 Appendix).

| Species | $\boldsymbol{r}_{\boldsymbol{max}}$ | $\boldsymbol{K}$, Ind. Km^-2^ | Length of study | Location | Method |
| --- | --- | --- | --- | --- | --- |
| **C.callipygus^1^** | 0.6^C&K^ | - | 2 months | Mossapoula, CAR* | 76 net hunts, N=24 |
| **C.callipygus^2^** | 0.07-0.3^C^ | - | - | Bayanga, CAR* | N=36, some parameters are from literature or personal communications |
| **C.callipygus^3^** | 0.5^C&K^ | 13.3-15.5 | - | Near Makokou, Gabon | Study site=80ha; home ranges used to estimate density |
| **C.callipygus^4^** | - | 7±1.8  ($\mu\pm se)$ | March 1981 - May 1983 | Kapituri, near Epulu, Ituri Forest, DRC** | 25 net drive counts |
| **C.callipygus^5^** | 0.29^C^ | - | - | - | From data in captivity |
| **C.callipygus^7^** | 0.51^C&K^ | 6.7 | March 1988 - December 1990 | Near Makokou, Gabon | 12 surveys, N=7 |
| **C.callipygus^11^** | - | 10.7 | - | - | - |
| **C.dorsalis^1^** | 0.55^C&K^ | - | 2 months | Mossapoula, CAR* | 76 net hunts, N=40 |
| **C.dorsalis^2^** | 0.05-0.3^C^ | - | - | Bayanga, Central African Republic | N=7, some parameters are from literature or personal communications |
| **C.dorsalis^3^** | 0.51^C&K^ | 7.5-8.7 | - | Near Makokou, Gabon | Study site=80ha; home ranges used to estimate density |
| **C.dorsalis^4^** | - | 7±1.8  ($\mu\pm se)$ | March 1981 - May 1983 | Kapituri, near Epulu, Ituri Forest, DRC** | 25 net drive counts |
| **C.dorsalis^5^** | 0.29^C^ | - | - | - | From data in captivity |
| **C.dorsalis^7^** | 0.49^C&K^ | 5.8 | March 1988 - December 1990 | Near Makokou, Gabon | 12 surveys, N=8 |
| **C.dorsalis^8^** | 0.2^C^ | - | 12 months | Bioko and Rio Muno, Equatorial Guinea | Reproduction estimates derived from Payne (1992), age of last reproduction substituted by max recorded longevity |
| **C.dorsalis^9^** | - | 1.9 (SD=1.41) | 6 months | Lenda, Ituri Forest, DRC** | 40 net drive counts |
| **C.dorsalis^9^** | - | 2.7 (SD=1.41) | 4 months | Edoro, Ituri Forest, DRC** | 40 net drive counts |
| **C.dorsalis^11^** | - | 7.1 | - | - | - |
| **C.monticola^1^** | 0.87^C&K^ | - | 2 months | Mossapoula, CAR* | 76 net hunts, N=440 |
| **C.monticola^2^** | 0.12-0.3^C^ | - | - | Bayanga, CAR* | N=38, some parameters are from literature or personal communications |
| **C.monticola^4^** | - | 13.6±1.6  ($\mu\pm se)$ | March 1981 - May 1983 | Kapituri, near Epulu, Ituri Forest, DRC* | 25 net drive counts |
| **C.monticola^5^** | 0.29^C^ | - | - |  | From data in captivity |
| **C.monticola^6^** | 0.39^C^ | - | 4 months | - | Reproduction estimates from literature |
| **C.monticola^7^** | 0.87^C&K^ | 30.8 | March 1988 - December 1990 | Near Makokou, Gabon | 12 surveys, N=44 |
| **C.monticola^8^** | 0.49^C^ | - | 12 months | Bioko and Rio Muno, Equatorial Guinea | Reproduction estimates derived from Payne (1992), age of last reproduction substituted by max |
| **C.monticola^9^** | - | 20.6 (SD=3.73) | 4 months | Edoro, Ituri Forest, Zaire | 40 net drive counts |
| **C.monticola^9^** | - | 10.2 (SD=3.62) | 6 months | Lenda, Ituri Forest, Zaire | 40 net drive counts |
| **C.monticola^10^** | - | 62-78 | 16 months (in 1971, 1972, 1973 and 1975) | Near Makokou, Gabon | 74 ha, capture-recapture |
| **C.monticola^11^** |  | 70 |  |  | - |
| **C.monticola^12^** | 0.85^C&K^ | - | 28 months between 1980 and 1984 | Nera Makokou, Gabon | Capture-recapture |
| **C.monticola^13^** | - | 61 | March 1983 | Ituri Forest, Congo-Zaire | 500x4m line transects |

^1^Noss [14]; ^2^Noss [27]; ^3^Feer [85]; ^4^Koster and Hart [86]; ^5^Noss [3]; ^6^Fitzgibbon, Mogaka and Fanshawe [26]; ^7^Lahm [33]; ^8^Fa et al., [25]; ^9^Hart [34]; ^10^DuBost [87], ^11^Feer (1996) quoted in Van Vliet and Nasi [16], ^12^DuBost [88]; ^13^Wilkie and Finn [11]

* Central African Republic

**Democratic Republic of Congo

S1 Fig. Sample densities for population growth rate, $\boldsymbol{r}_{\boldsymbol{max}}$ , and carrying capacity, $\boldsymbol{K}$. The values were used to inform our harvesting models (i.e. Prior belief) as a result of sampling from a log-normal distribution (natural logarithm), for a.) *C.callipygus*; b.) *C. dorsalis*; and c.) *C.monticola*. Field data is represented by red dots with sample sizes as follows: a.) $\boldsymbol{n}_{\boldsymbol{r}_{\boldsymbol{max}}}\boldsymbol{=5;}\boldsymbol{n}_{\boldsymbol{K}}\boldsymbol{=4}$; b.) $\boldsymbol{n}_{\boldsymbol{r}_{\boldsymbol{max}}}\boldsymbol{=6;}\boldsymbol{n}_{\boldsymbol{K}}\boldsymbol{=6}$; and c.) $\boldsymbol{n}_{\boldsymbol{r}_{\boldsymbol{max}}}\boldsymbol{=7;}\boldsymbol{n}_{\boldsymbol{K}}\boldsymbol{=7}$.





S1 Appendix. Detailed instructions for executing our method.

Yields from harvesting are estimated as follows:

(10)

$$Y\left( \varphi,N \right)=q\times\varphi\times N$$

$$Y\left( h \right)=h$$

where $N$ is the population size (follows the Beverton-Holt model, see below), $\varphi$ and $h$ are the harvest rates and $q$ is the catchability coefficient measuring the efficiency of each unit of hunting effort (equal to 1 for the purposes of this study).

We assume that animals are harvested at the end of each time step. The Beverton-Holt model has no age structure, and so the model assumes implicitly that every animal, including the newborns, can be extracted.

Using the Beverton-Holt model, the number of animals $N_{t}^{pre-harvest}$ at time $t$ before harvesting is applied:

$N_{t}^{pre-harvest}=\frac{r_{t}\times N_{t-1}^{post-harvest}}{(1+\frac{r_{t}-1}{K})\times N_{t-1}^{post-harvest}}$

(11)

where $N_{t-1}^{post-harvest}$ is the number of animals that survived harvesting in the previous time step, and $r_{t}$ is the intrinsic rate of natural increase at time $t$, sampled from a log-normal distribution as described by (3).

Total losses to harvesting, or yield ($Y_{t}$) at time $t:$

$Y_{t}=N_{t}^{pre-harvest}-N_{t}^{post-harvest}$

(12)

If every animal in the population has been extracted in the previous time step (i.e., no animals remain), the number of animals at time $t$, $N_{t}^{pre-harvest}$reverts to 0.

Under the constant proportional harvesting policy, the yield $Y_{t}$ depends upon the number of animals present at time $t$, and the harvest rate, $\varphi$. Under the constant quota-based policy, the yield $Y_{t}$ depends upon the target quota, $h$ only. The number of animals $N_{t}^{post-harvest}$ that remain in the population after harvesting at time $t$, is the higher of 0 (no animals survive to the next time step) and the number of animals after a proportion $\varphi$ or target quota $h$ of animals has been extracted.

$N_{t}^{post-harvest}=max(0,N_{t}^{pre-harvest}-\varphi\times N_{t}^{pre-harvest})$

(13)

$$N_{t}^{post-harvest}=max(0,N_{t}^{pre-harvest}-h)$$

To examine the impact of harvesting intensity, we carry out different $m$-year simulations (100, 50-, 20-and 5-year), each with a different harvest rate $\varphi$ or $h$. We calculate yield $Y_{t}$as an average of $m$ time steps. The rate of harvesting remains constant throughout the harvesting period of $m$ time steps.

Each simulation has the same harvest rate$(\varphi\mathrm{or}h$) but is subject to different parameter values, with additional variability introduced by stochastic growth rates (due to environmental stochasticity, see (2)). Environmental stochasticity was present in simulations without parameter uncertainty and with parameter uncertainty. From a technical perspective, the differences between simulations for a given harvest rate result from selecting different population parameter samples $(r_{max},K,r,D)$ from a random number generator.

We use discrete time formulation rather than continuous: as long as there are no substantial fluctuations in population dynamics within a year, population growth per year is assumed to be a reasonable approximation. Similarly, we consider harvesting a set number of animals per year (rather than continuously) a reasonable approximation of the real-life processes.

Dealing with low sample sizes using chi-squared $(\chi^{2})$ distribution

Because of the low number of estimates ($4\leq n\leq7$) for parameters in our dataset (i.e. observed parameter estimates), we were less confident that we were able to capture the true parameter values. To ensure that we included less likely harvesting scenarios we used the chi-squared distribution $(\chi^{2})$ to estimate confidence intervals for standard deviations based on sample standard deviations (i.e. the observed standard deviations that informed the parameter distributions). We constructed the confidence intervals for the standard deviation using the $\chi^{2}$-squared distribution at 95% confidence level with $n-1$ degrees of freedom:

$\sqrt{\frac{(n-1)s^{2}}{\chi_{\alpha/2}^{2}}}\leq\sigma\leq\sqrt{\frac{(n-1)s^{2}}{\chi_{1-\alpha/2}^{2}},}$

(14)

where $s$ is the sample standard deviation for each duiker species in our dataset, $\alpha$ is the significance level (=0.05) and $\chi^{2}$ is the critical value found from the table of $\chi^{2}$ values. For example, for Peters’ duiker ($n=5)$ the $\chi_{0.025,4}^{2}=0.484$ and the confidence intervals for standard deviation of ${ln(r}_{max})$ are $0.179\leq ln(\sigma)\leq0.86$. We use the upper tail (the higher value) as an estimate of standard deviation.

S2 Appendix. Intrinsic rate of natural increase $\boldsymbol{r}_{\boldsymbol{max}}$: Cole’s and Caughley and Krebs formulae.

The intrinsic rate of natural increase in studies in our dataset was estimated using either Cole’s [39], or Caughley and Krebs [40] formula.

Following Cole’s formula, intrinsic rate of natural increase $r_{max}$ was estimated using:

(15)

$$1=e^{{-r}_{max}}+{be}^{{-r}_{max}}a-be^{{-r}_{max}}(w+1)$$

where $a$ is the age at first reproduction, $b$ is the annual birth rate of female offspring and $w$ – age at last reproduction. Main criticisms of Cole’s formula is that mortality before the age of last reproduction ($w)$ is assumed to be zero for both adults and juveniles. In addition, because the population information is unknown for some of duiker species, the same values for reproduction parameters have been often used for blue C. monticola, red (C. callipygus, C. dorsalis, C. nigrifrons, C. leucogaster, C. ogylbi) or yellow C. sylvicultor duikers [16].

Because of poor knowledge of duiker mortality and fecundity [16], some authors [14, 85, 89] use Caughley and Kreb’s formula to estimate $r_{max}$:

$r_{max}=1.5P^{(-0.36)}$

(16)

which only relies on assumptions about $P$ - the mean population weight in kg. To take account of the age structure of the population, some studies used the percentage of mean weight of an adult duiker. Noss [27] used the actual weight of carcasses sold on markers.

S3 Appendix. Actual bushmeat offtakes for the three duiker species across the Congo basin. Estimates are based on the total bushmeat exploitation estimates (bushmeat eaten by country) by (a) Wilkie and Carpenter [38] and (b) Fa, Currie and Meeuwig [42]. We used forest area [38] to calculate biomass km^-2^ year^-1^. The percentage exploitation of each of the three duiker species was estimated using bushmeat census data [25, 90, 91]. We used mean body masses in Table 1 to convert biomass to the number of animals harvested. CAR = Central African Republic, DRC=Democratic Republic of Congo.

**(a)**

| **Country** | **Forest area (km^2^)** | **Bushmeat eaten (kg year^-1^)** | **Bushmeat eaten (kg km^-2^ year^-1^)** | **% of total biomass exploitation** | | | **Biomass exploitation (kg km^-2^ year^-1^)** | | | **Number harvested (animals km^-2^ year^-1^)** | | | **Adapted from** |
| --- | --- | --- | --- | --- | --- | --- | --- | --- | --- | --- | --- | --- | --- |
|  |  |  |  | **Peters** | **Bay** | **Blue** | **Peters** | **Bay** | **Blue** | **Peters** | **Bay** | **Blue** |  |
| Cameroon | 155330 | 78077172 | 503 | 0.0045 | 0.06 | 0.1 | 2.3 | 30.2 | 50.3 | 0.14 | 1.68 | 10.88 | [92] |
| CAR | 52236 | 12976507 | 248 | 0.09 | 0.19 | 0.63 | 22.4 | 47.2 | 156.5 | 1.38 | 2.62 | 33.88 | [90] |
| CAR | 52236 | 12976507 |  |  |  |  |  |  |  | 1.00 | 1.60 | 17.30 | [14] |
| DRC | 1190737 | 1067873491 | 897 |  | 0.06 | 0.06 |  | 53.8 | 53.8 |  | 2.99 | 11.65 | [91] |
| Equatorial Guinea | 17004 | 9762838 | 574 |  | 0.2 | 0.22 |  | 114.8 | 126.3 |  | 6.38 | 27.34 | [25], Rio Muni |
| Equatorial Guinea | 17004 | 9762838 | 574 |  |  | 0.03 |  |  | 17.2 |  |  | 3.73 | [25], Bioko |
| Gabon | 227500 | 11380598 | 50 |  |  |  |  |  |  |  |  |  |  |
| Republic of Congo | 213400 | 16325305 | 77 |  |  |  |  |  |  |  |  |  |  |
|  |  |  |  |  |  |  |  | **Median** |  | **1.00** | **2.62** | **14.47** |  |
|  |  |  |  |  |  |  |  | **S.d.** |  | **0.63** | **1.95** | **11.25** |  |

(b)

| **Country** | **Forest area (km^2^)** | **Bushmeat eaten (kg year^-1^)** | **Bushmeat eaten (kg km^-2^ year^-1^)** | **% of total biomass exploitation** | | | **Biomass exploitation (kg km^-2^ year^-1^)** | | | **Number harvested (animals km^-2^ year^-1^)** | | | **Adapted from** |
| --- | --- | --- | --- | --- | --- | --- | --- | --- | --- | --- | --- | --- | --- |
|  |  |  |  | **Peters** | **Bay** | **Blue** | **Peters** | **Bay** | **Blue** | **Peters** | **Bay** | **Blue** |  |
| Cameroon | 155330 | 233963266 | 1506 | 0.0045 | 0.06 | 0.1 | 2.3 | 30.2 | 50.3 | 0.42 | 5.02 | 32.60 | [92] |
| CAR | 52236 | 48821704 | 935 | 0.09 | 0.19 | 0.63 | 22.4 | 47.2 | 156.5 | 5.19 | 9.87 | 127.45 | [90] |
| CAR | 52236 | 48821704 |  |  |  |  |  |  |  | 1.00 | 1.60 | 17.30 | [14] |
| DRC | 1190737 | 1665972491 | 1399 |  | 0.06 | 0.06 |  | 53.8 | 53.8 |  | 4.67 | 18.17 | [91] |
| Equatorial Guinea | 17004 | 12937737 | 761 |  | 0.2 | 0.22 |  | 114.8 | 126.3 |  | 8.46 | 36.23 | [25], Rio Muni |
| Equatorial Guinea | 17004 | 12937737 | 761 |  |  | 0.03 |  |  | 17.2 |  |  | 4.94 | [25], Bioko |
| Gabon | 227500 | 49069902 | 216 |  |  |  |  |  |  |  |  |  |  |
| Republic of Congo | 213400 | 189234900 | 887 |  |  |  |  |  |  |  |  |  |  |
|  |  |  |  |  |  |  |  | **Median** |  | **1.00** | **5.02** | **25.39** |  |
|  |  |  |  |  |  |  |  | **S.d.** |  | **2.60** | **3.28** | **44.58** |  |

S4 Appendix. Our method of combining field data with the harvest model.

The observed values for $r_{max}$ and $K$ (S1 Table) are used to estimate the mean (${\overset{^}{r}}_{max}$, $\overset{^}{K}$) and the corresponding uncertainty (${\overset{\sim}{r}}_{max}$ and $\overset{\sim}{K}$ ) for each of these population parameters; these become ‘Prior Belief’. We then use this Prior Belief about the true population parameters to inform the harvest model that also includes yearly changes in environmental conditions (‘Yearly Environmental Stochasticity’), to estimate yields and survival probability for the three duiker species using two constant harvesting strategies under parameter uncertainty. These predictions can then be used by bushmeat practitioners (‘Stakeholder Groups’) to guide their choice of harvest rate (subject to attitude to risk) and their expectations of harvesting outcomes.

The method assumes only two prerequisites: a model formulation that is believed to be appropriate for simulating the dynamics of population size and yield through time, given harvesting; and explicit prior beliefs, based on field data, on the parameters of that model. The method could be applied wherever these prerequisites are available. Given the prerequisites, the method uses ensemble modelling to estimate the probability distributions on population extinction, and yield, for different harvesting levels calculated over different time periods. These distributions can then be fed into a risk-based decision-making process, to help set actual harvesting levels. In common with all methods employing ecological modelling [47, 61, 93], the method ignores many key ecological complexities that may affect populations and yield in reality.

S2 Fig. Peters’ duiker *C.callipygus*. Survival probability (with 95% confidence intervals) and estimated yields with parameter uncertainty and environmental stochasticity under (a.) constant quota-based and (b.) proportional harvesting over four time horizons (5, 20, 50 and 100 years). Dotted vertical lines represent harvest rates above which harvesting is expected to drive over 10% of duiker species to extinction over the harvesting horizon.





S3 Fig. Bay duiker *C.dorsalis*. Survival probability (with 95% confidence intervals) and estimated yields with parameter uncertainty and environmental stochasticity under (a.) constant quota-based and (b.) proportional harvesting over four time horizons (5, 20, 50 and 100 years). Dotted vertical lines represent harvest rates above which harvesting is expected to drive over 10% of duiker species to extinction over the harvesting horizon.





S4 Fig. Blue duiker *C.monticola*. Survival probability (with 95% confidence intervals) and estimated yields with parameter uncertainty and environmental stochasticity under (a.) constant quota-based and (b.) proportional harvesting over four time horizons (5, 20, 50 and 100 years). Dotted vertical lines represent harvest rates above which harvesting is expected to drive over 10% of duiker species to extinction over the harvesting horizon.

**
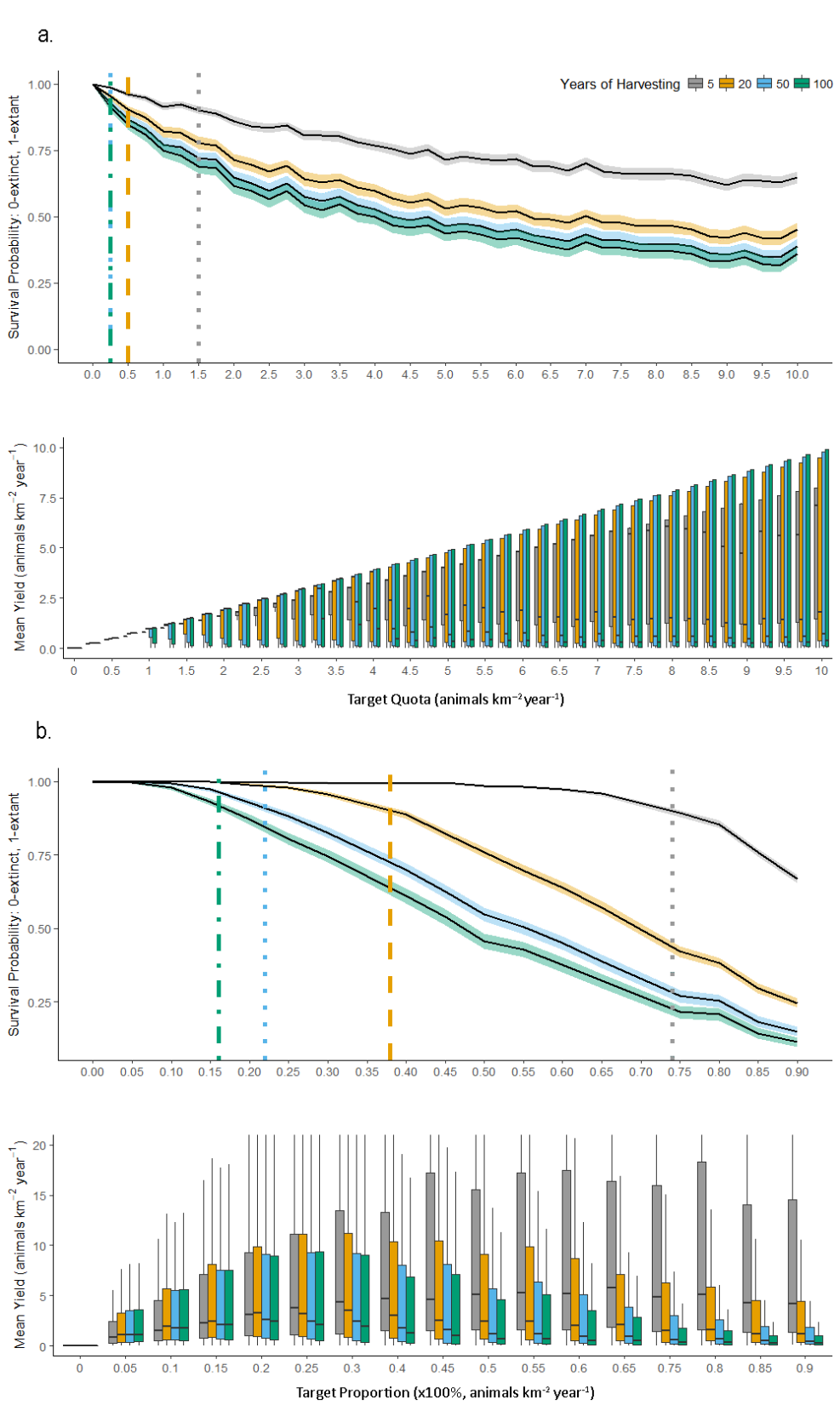
**

S5 Fig. Peters’ duiker *C.callipygus*. Estimated yields (animals km^-2^ year^-1^) from proportional harvesting over 25 years in 5-year increments without (a.) and with (b.) parameter uncertainty, with corresponding survival probabilities (in top-right corner of each rectangle).


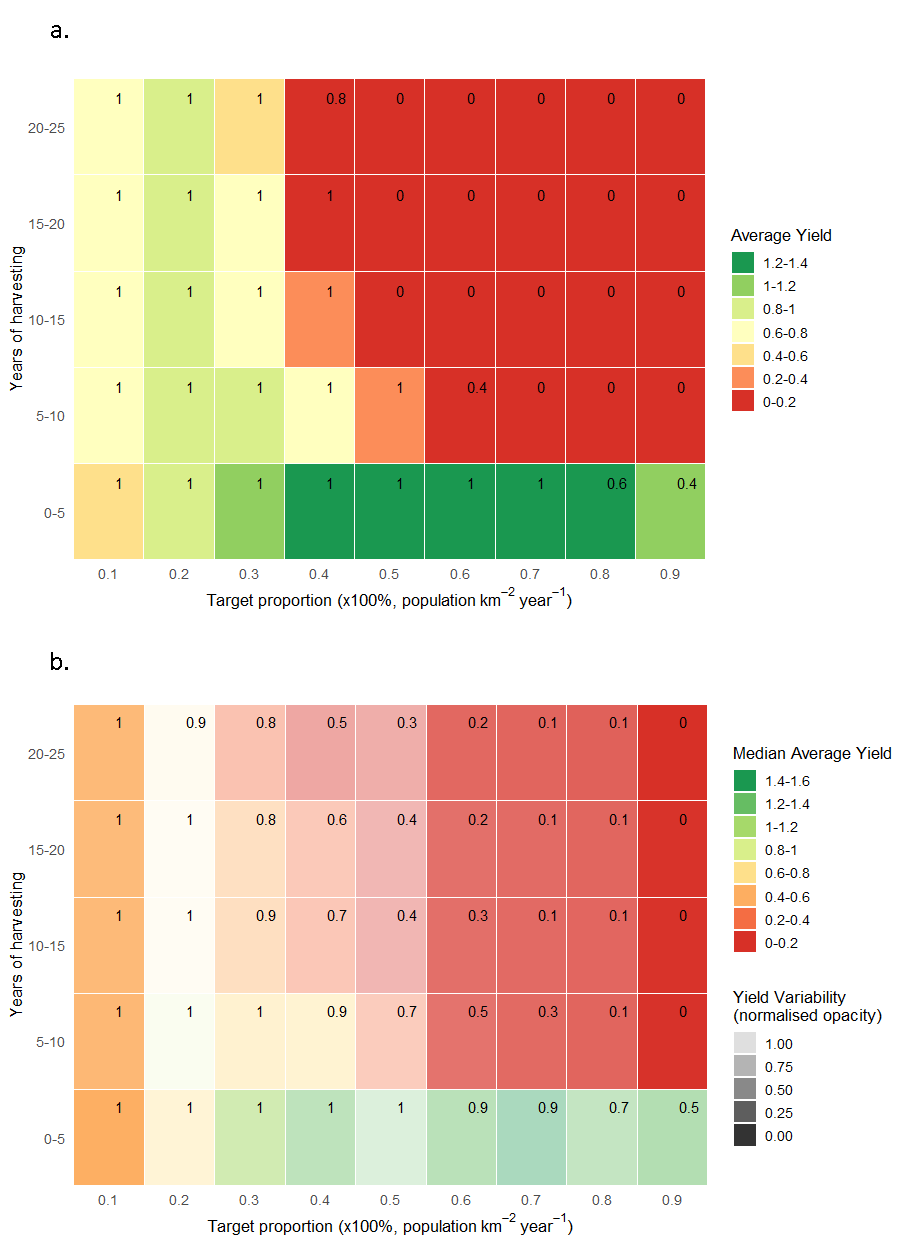


S6 Fig. Bay duiker *C.dorsalis.* Estimated yields (animals km^-2^ year^-1^) from proportional harvesting over 25 years in 5-year increments without (a.) and with (b.) parameter uncertainty, with corresponding survival probabilities (in top-right corner of each rectangle).

**
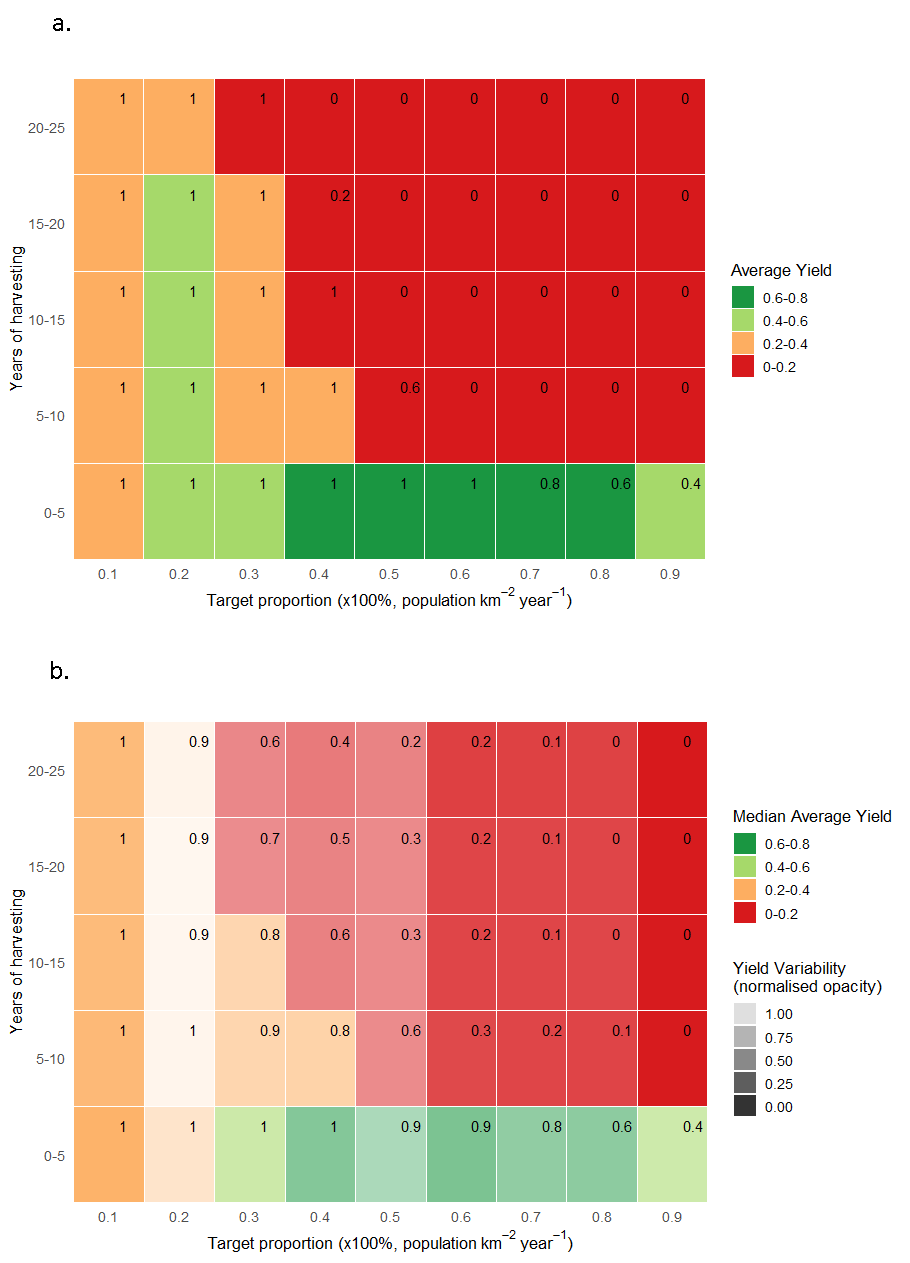
**

S7 Fig. Blue duiker *C.monticola*. Estimated yields (animals km^-2^ year^-1^) from proportional harvesting over 25 years in 5-year increments without (a.) and with (b.) parameter uncertainty, with corresponding survival probabilities (in top-right corner of each rectangle).

**
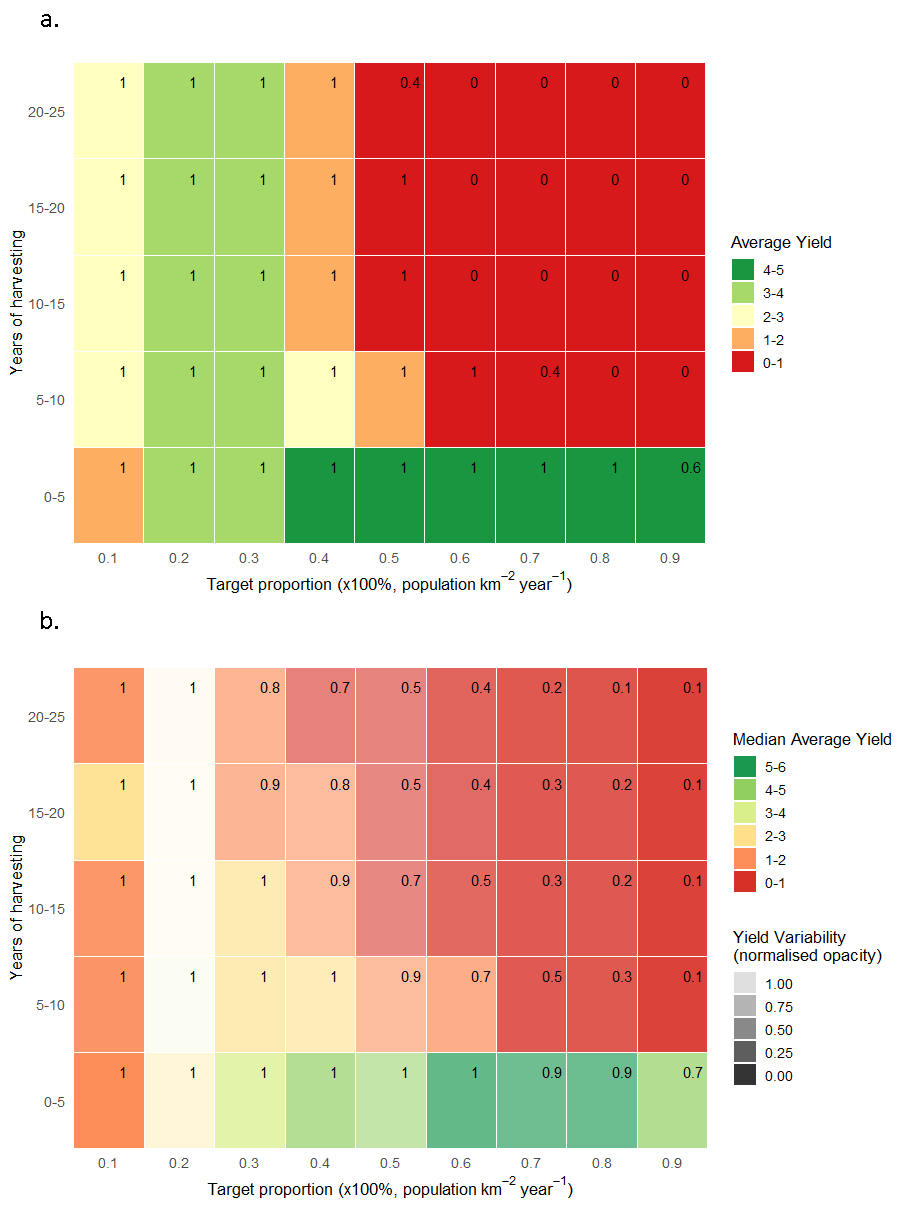
**

S8 Fig. A screenshot of our interactive online application. The application was built to support decision-making in bushmeat harvesting (for Peters’ duiker *Cephalophus callipygus*). Users can adjust population parameter values $\boldsymbol{r}_{\boldsymbol{max}}$ and $\boldsymbol{K}$ (as highlighted by red arrows) via sliders in the grey box on the left. The output includes average yields and survival probability. The red area on the plots indicates harvest rates that could drive over 10% of animal population to extinction over the duration of harvesting horizon (also user-defined). The app is hosted at <http://tinyurl.com/duikerantelope>. The map was made with Natural Earth. Free vector and raster map data @ naturalearthdata.com.


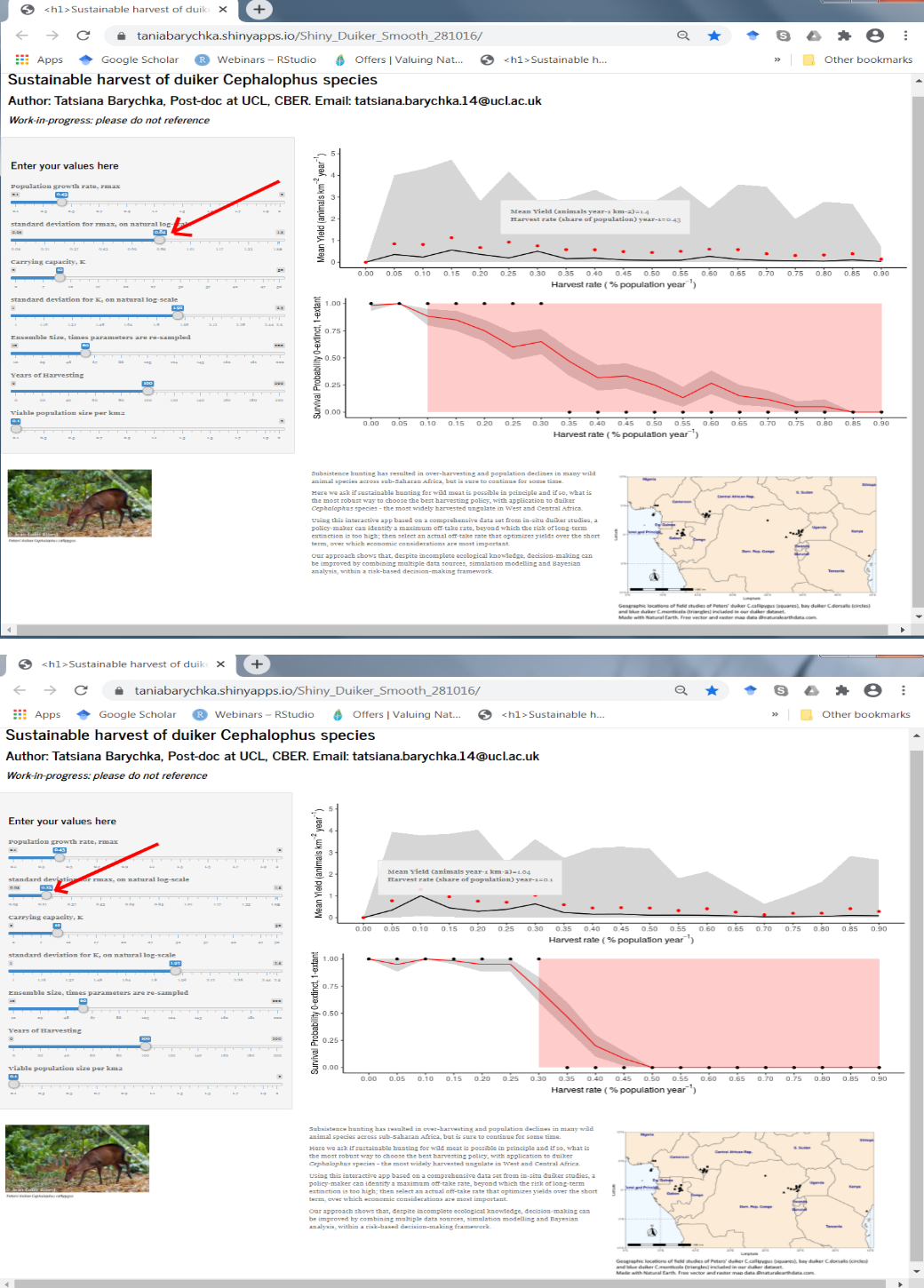


References

85. Feer F. Stratégies écologiques de deux espèces de Bovidés sympatriques de la forêt sempervirente africaine (Cephalophus callipygus et C. dorsalis): influence du rythme d/'activité. 1988.

86. Koster SH, Hart JA. Methods of estimating ungulate populations in tropical forests. African Journal of Ecology. 1988;26(2):117-26.

87. Dubost G. L ‘écologie et la vie sociale du Céphalophe bleu (Cephalophus monticola Thunberg), petit ruminant forestier africain. Zeitschrift für Tierpsychologie. 1980;54(3):205-66.

88. Dubost G. The size of African forest artiodactyls as determined by the vegetation structure. African Journal of Ecology. 1979;17(1):1-17.

89. Dethier M, Ghuirghi A. Etude de la chasse villageoise dans le secteur Ouest (route Mambélé-Ndélé) de la zone d'intervention du projet ECOFAC. Bangui (RCA), Ministère de l'Environnement, des Eaux, Forêts, Chasses et Pêches, ECOFAC. 2000.

90. Noss AJ. Duikers, cables, and nets: a cultural ecology of hunting in a central African forest. 1995.

91. van Vliet N, Nebesse C, Gambalemoke S, Akaibe D, Nasi R. The bushmeat market in Kisangani, Democratic Republic of Congo: implications for conservation and food security. Oryx. 2012;46(02):196-203.

92. Fa JE, Seymour S, Dupain JEF, Amin R, Albrechtsen L, Macdonald D. Getting to grips with the magnitude of exploitation: bushmeat in the Cross–Sanaga rivers region, Nigeria and Cameroon. Biological Conservation. 2006;129(4):497-510.

93. Phillips SJ, Anderson RP, Schapire RE. Maximum entropy modeling of species geographic distributions. Ecological Modelling. 2006;190(3-4):231-59.
